# Supplementary material for: A replication-linked mutational gradient drives somatic mutation accumulation and influences germline polymorphisms and genome composition in mitochondrial DNA
Source: Nucleic Acids Res. 2021 Oct 6;49(19):11103–18. doi: 10.1093/nar/gkab901 (PMC8565317; doi:10.1093/nar/gkab901)
Supplement: gkab901_Supplemental_Files [file gkab901_supplemental_files.zip › Supplementary Figures-revised.docx]

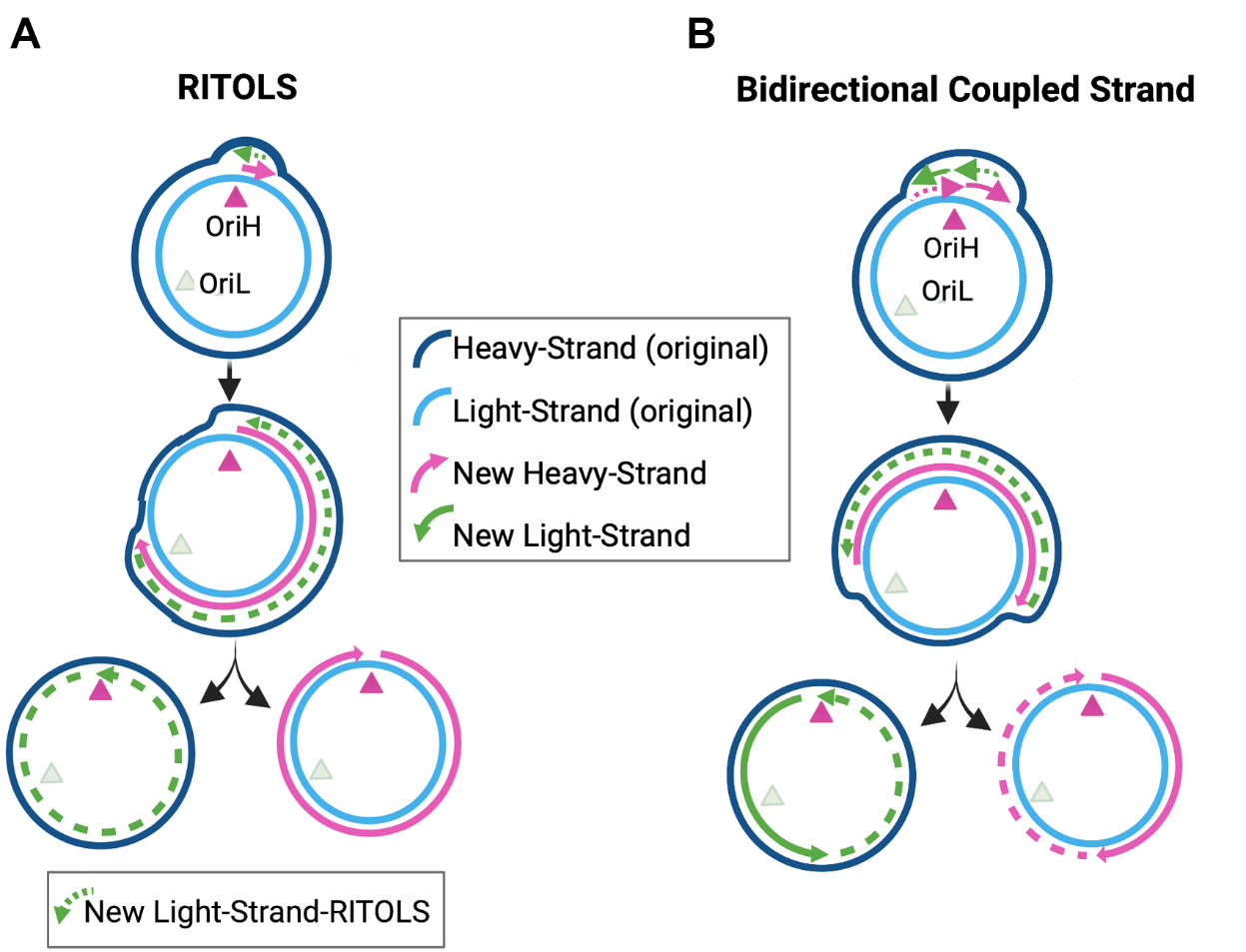


**Supplementary Figure S1.** Alternative replication models for mammalian mtDNA. **(A)** Schematic of RITOL model. **(B)** Schematic of strand-synchronous bidirectional replication model. Figure adapted from Lujan *et al*. and licensed under the CC BY 4.0 (1).

**Supplementary Figure S2.** Per gene analysis of G🡪A mutation frequencies in aged (26mo) ­­­kidney sorted by gene position. Mutation frequency was calculated by dividing the number of G🡪A mutations within the specified gene by the number of genes sequenced in the same gene. Order of magnitude of the mutation frequency is denoted in the upper left corner. Error bars denote standard deviation. Correlation determined by Spearman’s correlation.


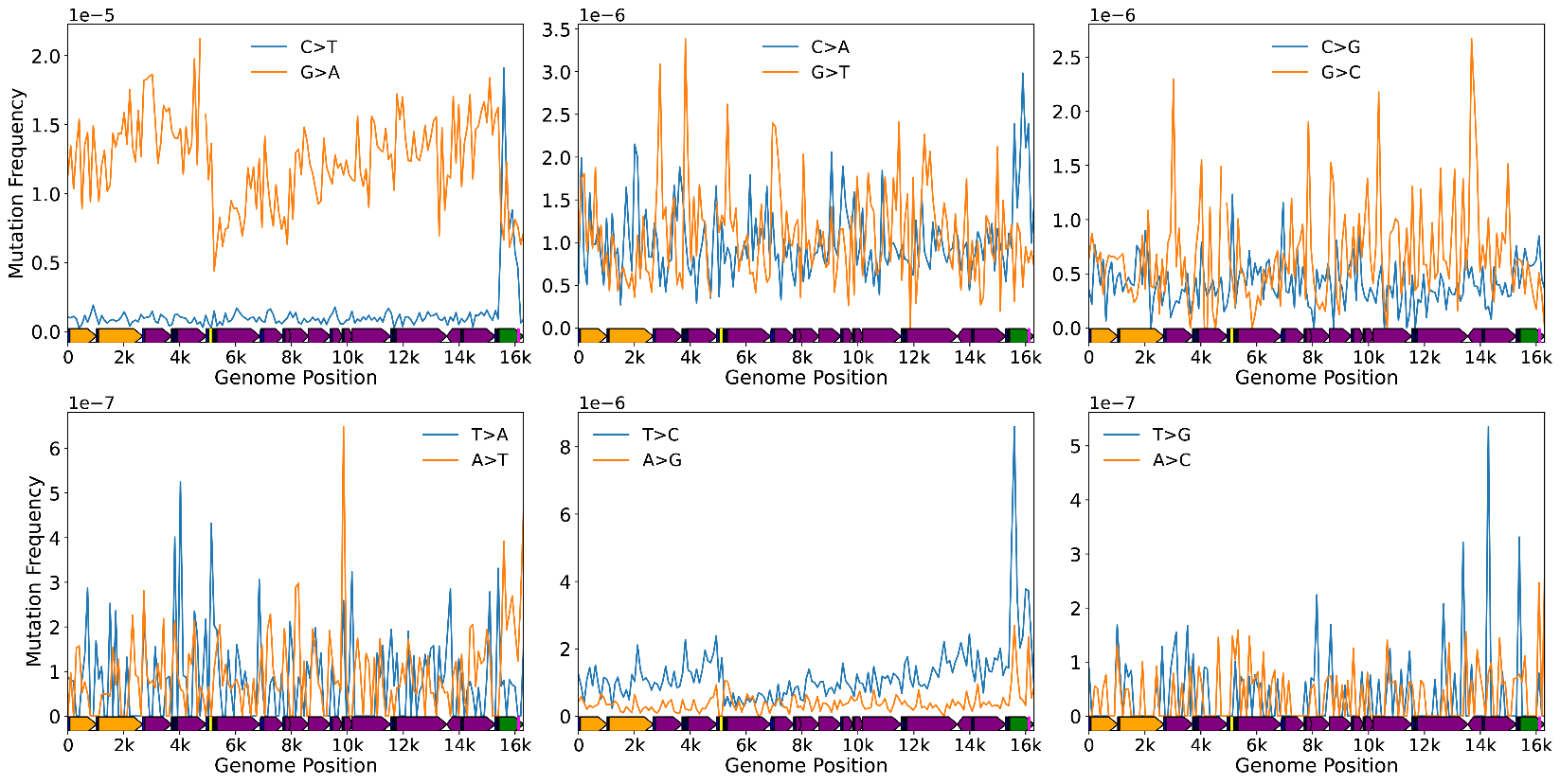


**Supplementary Figure S3.** Distribution of all 12 mutation classes across the wild-type mouse mtDNA. Mutations are reported as found on the L-strand. Complementary mutation types are grouped together in the same pane. Order of magnitude for the bin-specific mutation frequency for reciprocal types is located at the upper left of each pane. Mouse mtDNA structure and coordinates are shown on the x-axis and are the same for all panels (*orange*=rRNA gene*, purple*=protein coding*, dark blue*=tRNA gene*, green*=control region, *yellow*=Ori_L_, *magenta*=Ori_H_).

**
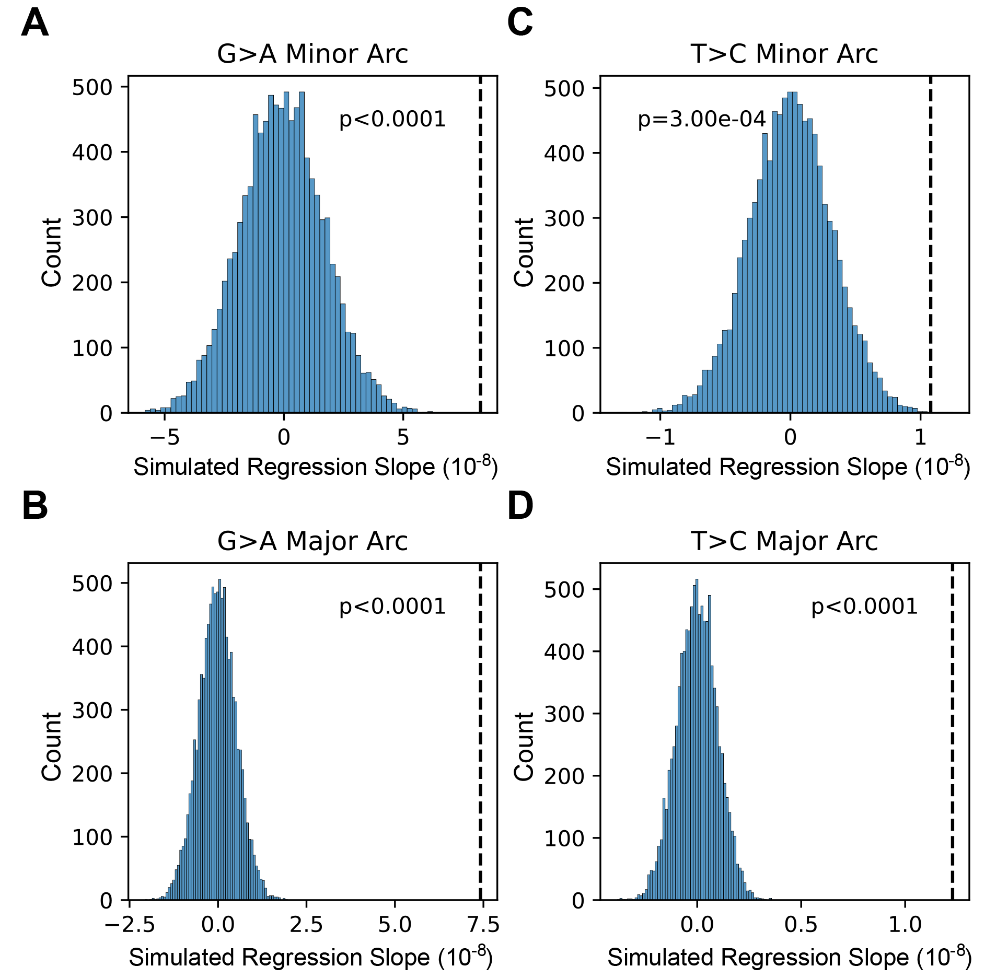
**

**Supplementary Figure S4.** Histogram of regression slopes of simulated somatic mutation frequencies across mouse mtDNA based on the weighted randomize distribution of observed mutations in 10,000 trials. Dotted vertical line represents observed slope reported in Supplementary Table S1. The p-value was calculated as the number of trials where the regression slope of the simulated data was greater than or equal to the observed regression slope divided by the number of trials (*i.e.* 10,000X).


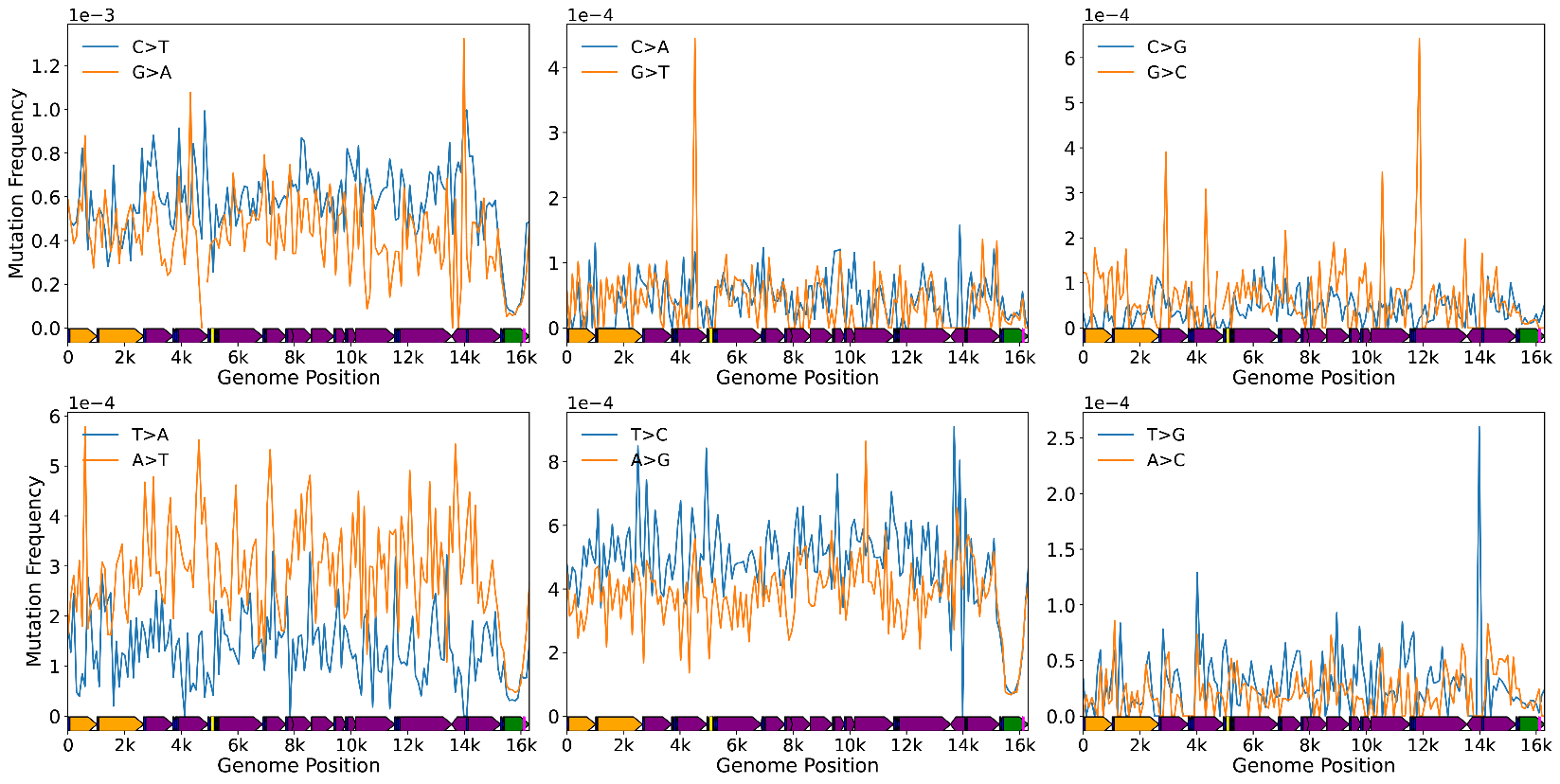


**Supplementary Figure S5.** Distribution of all 12 mutation classes across the Pol-γ^exo-^ mouse mtDNA. Mutations are reported as found on the L-strand. Complementary mutation types are grouped together in the same pane. Order of magnitude for the bin-specific mutation frequency for reciprocal types is located at the upper left of each pane. Mouse mtDNA structure and coordinates are shown on the x-axis and are the same for all panels (*orange*=rRNA gene*, purple*=protein coding*, dark blue*=tRNA gene*, green*=control region, *yellow*=Ori_L_, *magenta*=Ori_H_).

**
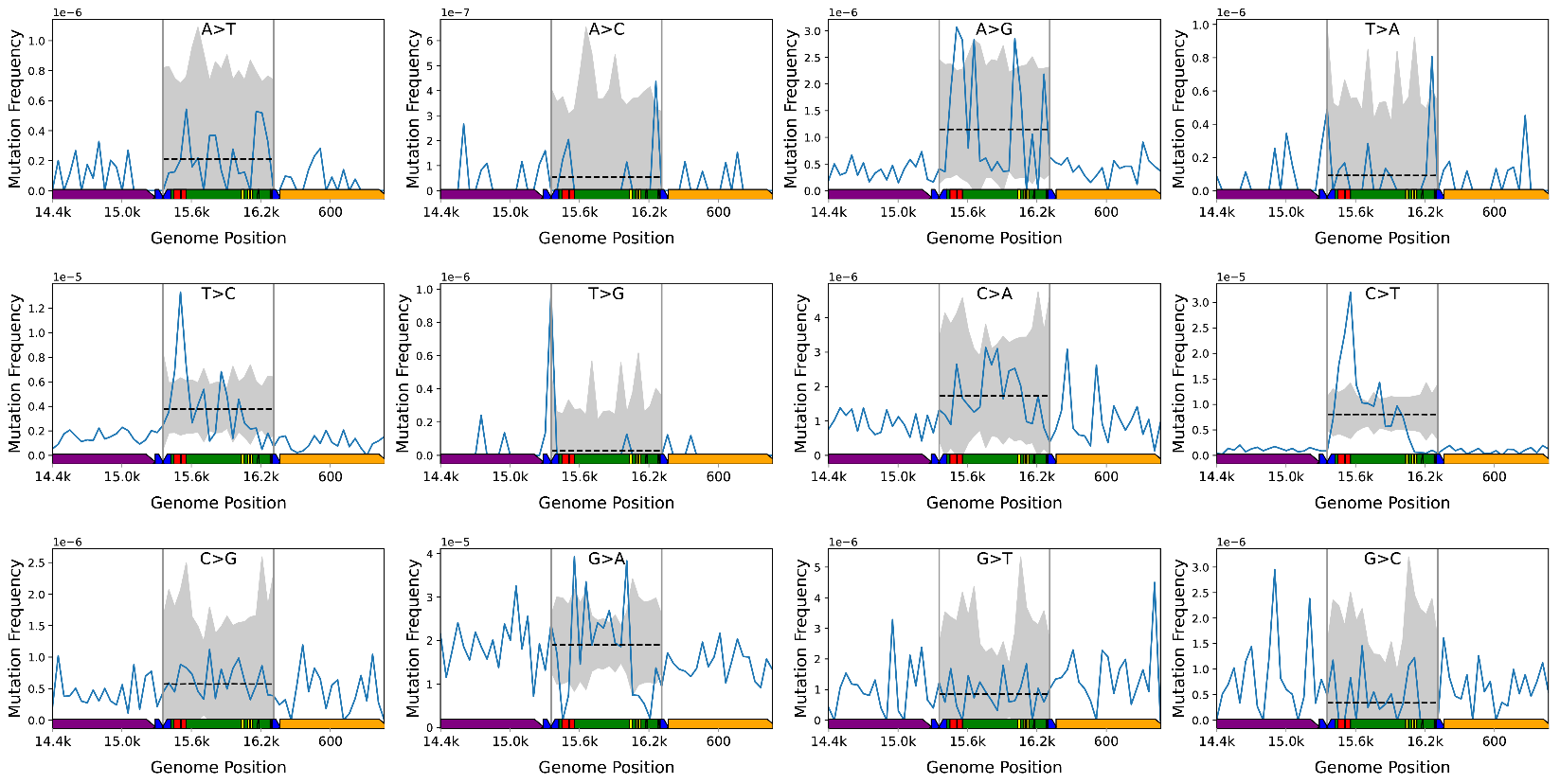
**

**Supplementary Figure S6.** Simulated distribution of mutations in the mouse mtDNA control region. The observed bin-specific mutation frequencies is reported as the blue line. The simulated bin-specific mean is reported as a dashed line. The corrected confidence interval (99.975%) of 100,000 simulations is shaded in gray. Any observed bin-specific mutation frequencies outside of the shaded region are over- or under-represented. Order of magnitude for each pane is located in the upper left corner. Vertical gray lines denote boundaries of the bins used for the simulation and are the nearest bin encompassing any sequence of the CR. Mouse mtDNA structure and coordinates are shown on the x-axis and are the same for all panels (orange=*mt-Rnr1* gene*, purple*=*Cytb* gene*, dark blue*=tRNA genes*, green*=control region, *red*=ETAS1&2; *yellow*=CSB1-3).

**
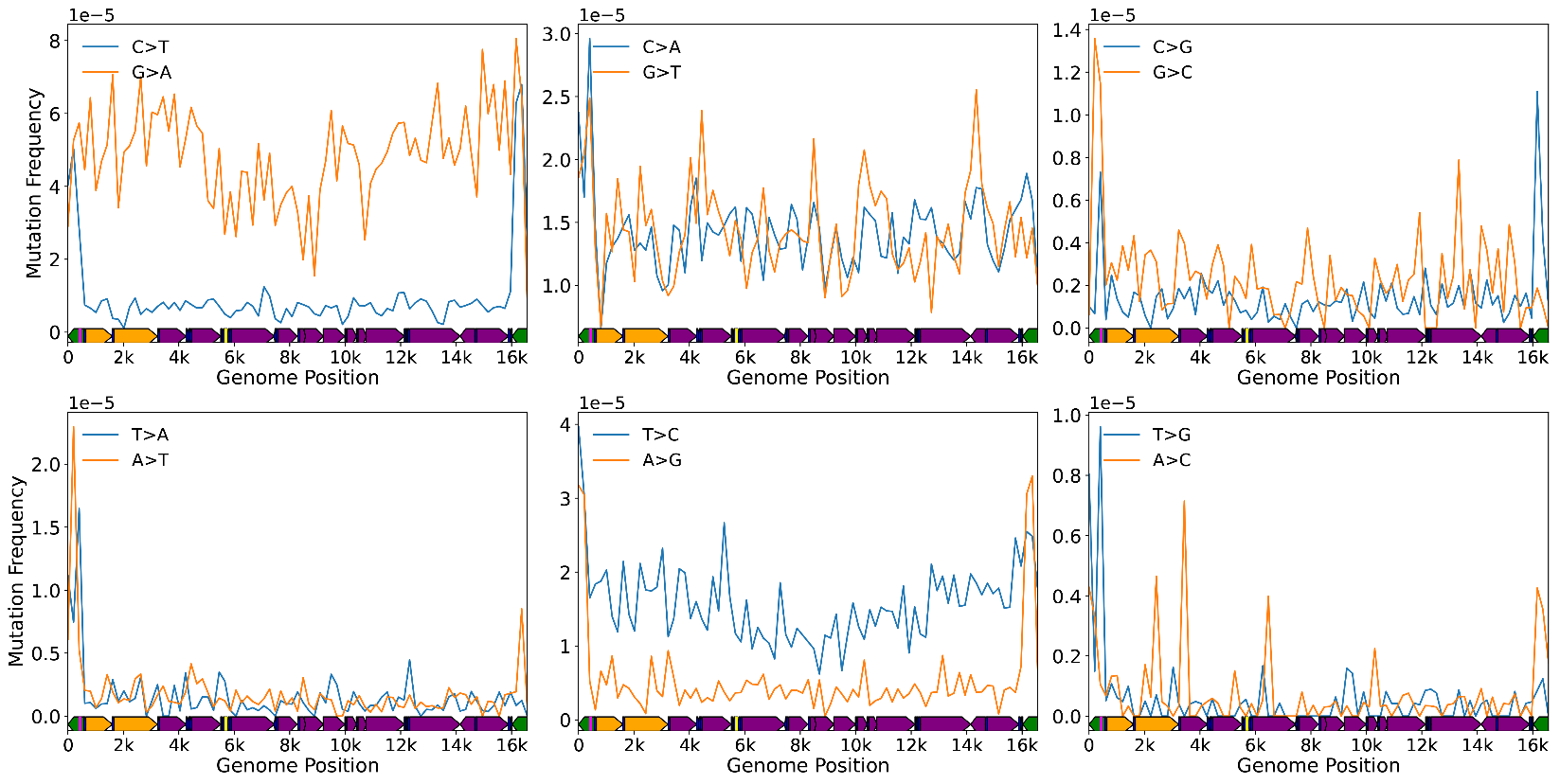
**

**Supplementary Figure S7.** Distribution of all 12 mutation classes across the human mtDNA. Mutations are reported as found on the L-strand. Complementary mutation types are grouped together in the same pane. Order of magnitude for the bin-specific mutation frequency for reciprocal types is located at the upper left of each pane. Human mtDNA structure and coordinates are shown on the x-axis and are the same for all panels (*orange*=rRNA gene*, purple*=protein coding*, dark blue*=tRNA gene*, green*=control region, *yellow*=Ori_L_, *magenta*=Ori_H_)

**
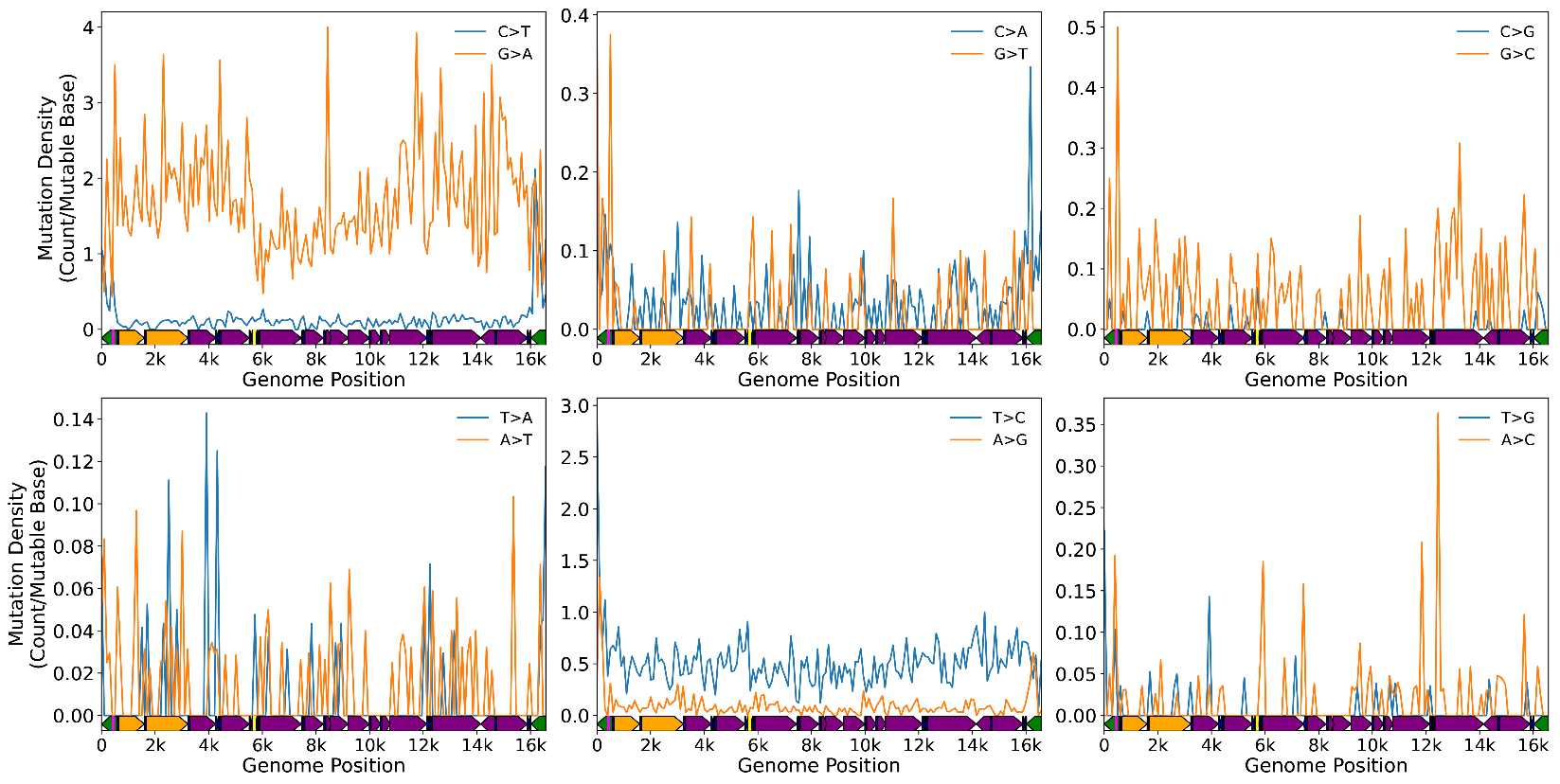
**

**Supplementary Figure S8.** Distribution of all 12 mutation classes across the human mtDNA as reported in the PCAWG data. Mutations are reported as found on the L-strand. Complementary mutation types are grouped together in the same pane. Human mtDNA structure and coordinates are shown on the x-axis and are the same for all panels (*orange*=rRNA gene*, purple*=protein coding*, dark blue*=tRNA gene*, green*=control region, *yellow*=Ori_L_, *magenta*=Ori_H_).


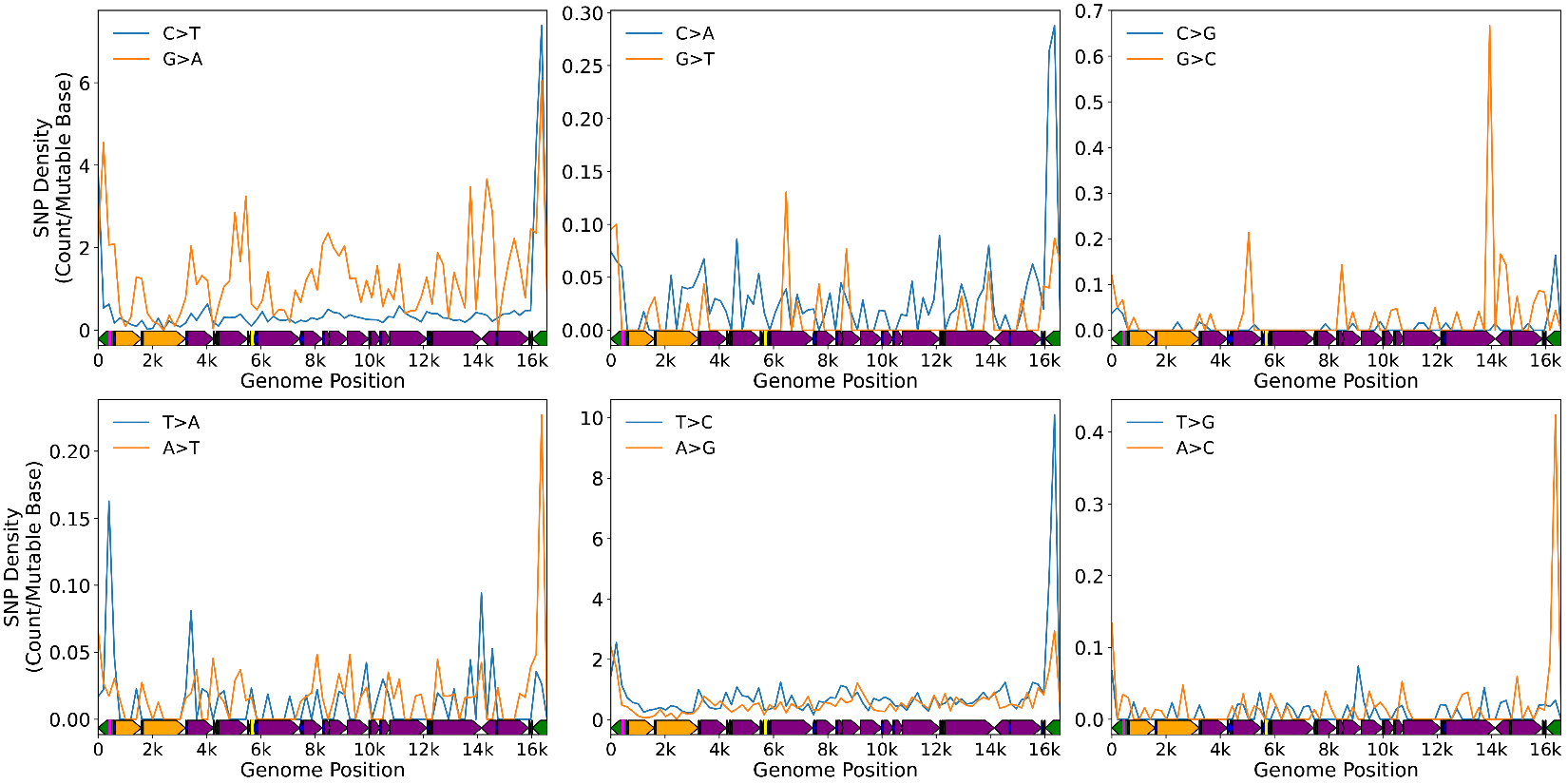


**Supplementary Figure S9.** Distribution of SNPs from all 12 mutation classes across the human mtDNA. Mutations are reported as found on the L-strand. Complementary mutation types are grouped together in the same pane. Data are taken direct from Supplemental Table 2 in Gu *et al.* (37). Human mtDNA structure and coordinates are shown on the x-axis and are the same for all panels (*orange*=rRNA gene*, purple*=protein coding*, dark blue*=tRNA gene*, green*=control region, *yellow*=Ori_L_, *magenta*=Ori_H_).


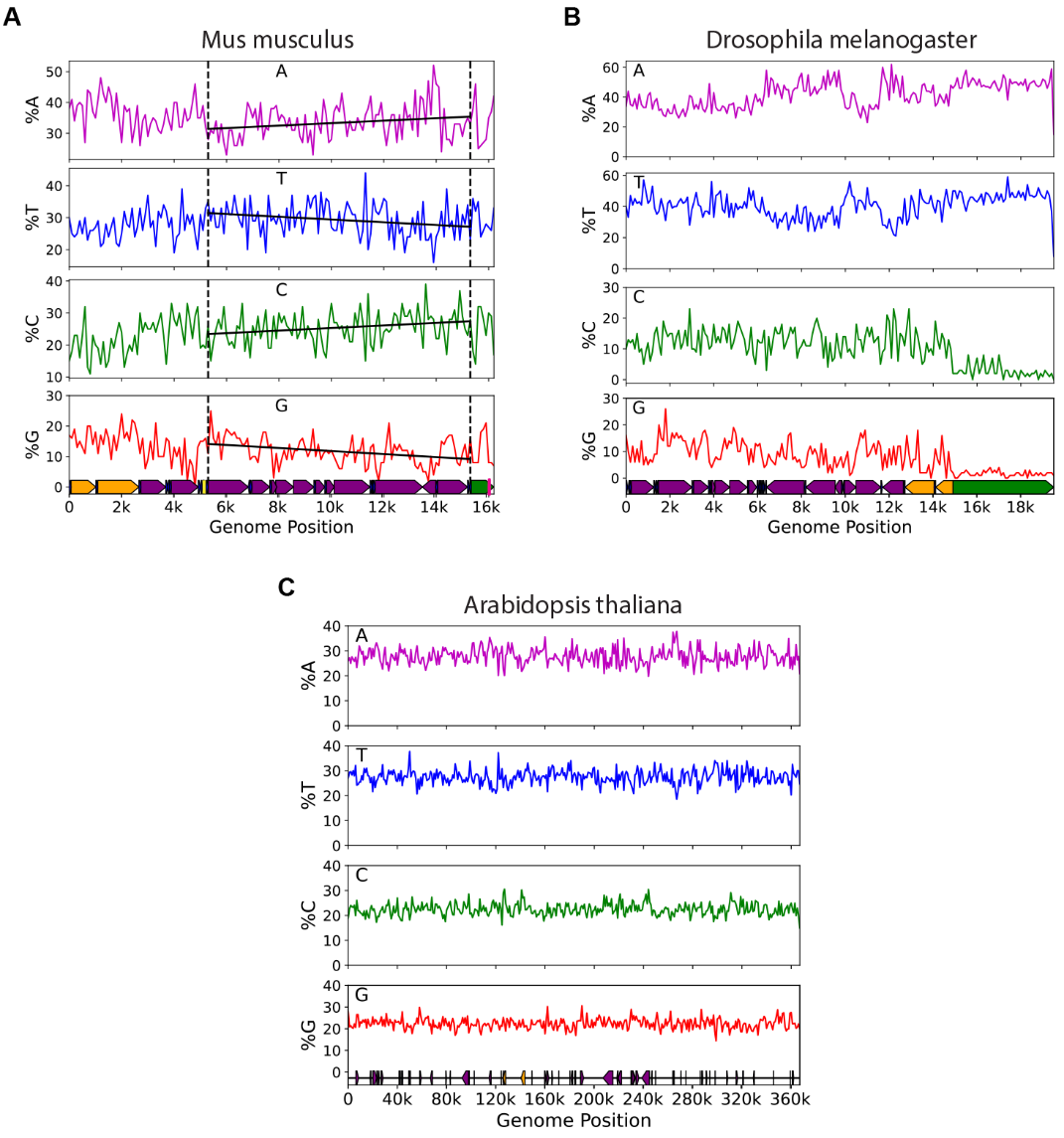


**Supplementary Figure S10. (A)** Base composition gradient as exemplified by the murine mtDNA. Vertical dashed lines delimit the major arc. Solid black lines are the best fit regression by robust linear regression. Slopes are significantly different from zero in all cases. **(B)** Base composition of *Drosophila melanogaster* mtDNA **(C)** Base composition of *Arabidopsis thaliana* mtDNA. Gene coloring: *orange*=rRNA gene*, purple*=protein coding*, dark blue*=tRNA gene*, green*=control region; *yellow*=Ori_L_, *magenta*=Ori_H_.

**
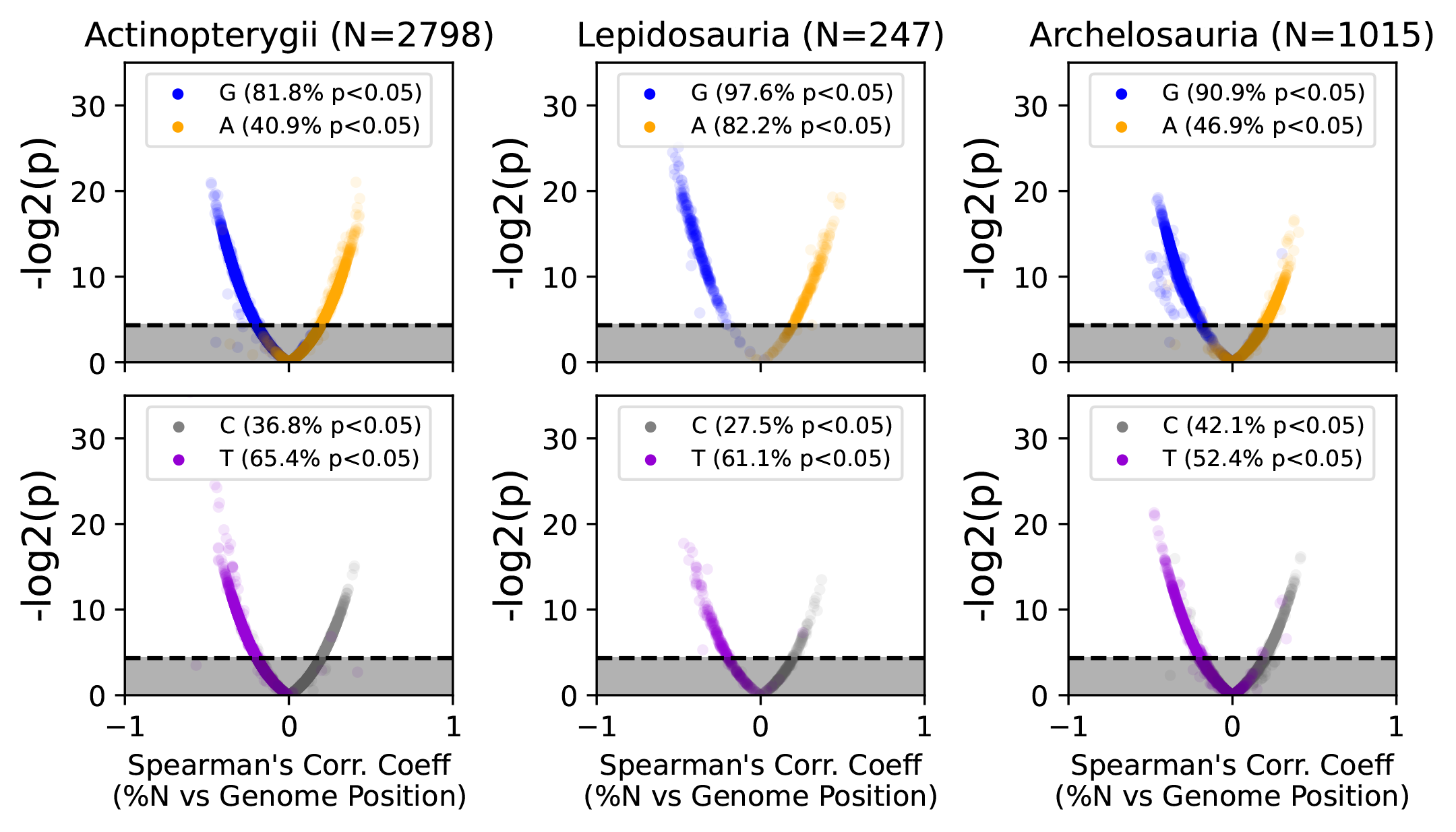
**

**Supplementary Figure S11. Volcano plot significant correlation between base composition and bin number across mtDNA sequences of diverse vertebrate species.** Base correlations are vertically grouped by taxonomic Class. Gray shaded area denotes non-significant correlation (p>0.05). Dotted line denotes p=0.05.


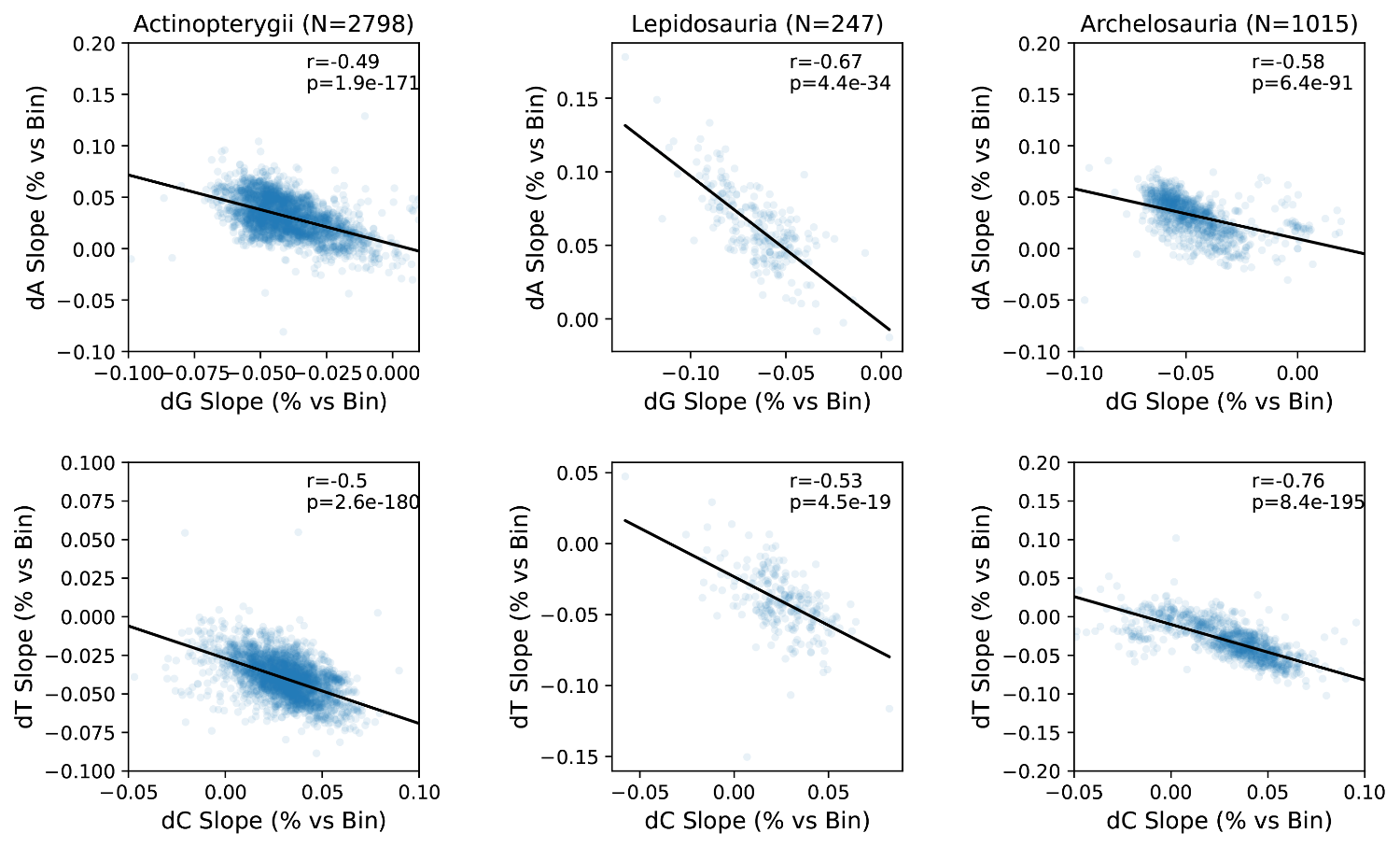


**Supplementary Figure S12. Correlation of slopes base composition vs number across mtDNA sequences of diverse vertebrate species.** Base correlations were determined by Spearman’s correlation and grouped vertically by taxonomic Class with dG vs dA on the top and dT vs dC on the bottom. Black line denotes best fit by a robust linear model as described in the methods.
